# Rosai-Dorfman disease as a diferential diagnosis of nasosinusal polyposis in children

**DOI:** 10.1590/S1808-86942012000300024

**Published:** 2015-10-14

**Authors:** Melissa A.G. Avelino, Thaís Gomes Abrahão Elias, Renata Martins Dayrel Rezende, Ana Paula Lindoso Lima, Alexandra Vilela Gonçalves

**Affiliations:** a PhD on ENT at UNIFESP-EPM; Associate Professor at PUC-GO (ENT at the Goiânia Children's Hospital and Associate Professor at PUC-GO).; b Medical Student at PUC-GO (Medical Student at PUC-GO).; c Medical Student at PUC-GO (Medical Student at PUC-GO).; d Medical Student at PUC-GO (Medical Student at PUC-GO).; e MD, Pediatric Hematologist (MD, Pediatric Hematologist). Hospital da Criança de Goiânia.

**Keywords:** diagnosis, histiocytosis, paranasal sinuses

## INTRODUCTION

Rosai-Dorfman disease (RDD) is characterized by primary proliferation of histiocytes in the lymph node sinuses^1^. It is a rare, benign, self-limiting proliferative condition^2^. Classical clinical manifestations include painless bilateral enlarged glands along the neck area. It may be associated with fever, weight loss, leukocytosis, altered inflammation test results, and positional hypergammaglobulinemia. The most frequently involved extranodal sites are the nasal cavities and the paranasal sinuses; their involvement leads to the onset of chronic high respiratory obstruction. Other authors have reported the involvement of various sites such as soft tissue, central nervous system, skin, eyeball, bones, kidneys, gastrointestinal tract, testicles, salivary glands, and respiratory tract^3^.

This case report aims to describe an atypical manifestation of RDD with primary involvement of the paranasal sinuses alone, unaccompanied by lymph node or adenomegaly; the case also reinforces the relevance of considering systemic disease when CT scans indicate nose and sinus polyposis in pediatric patients.

## CASE PRESENTATION

**Figure 1:**
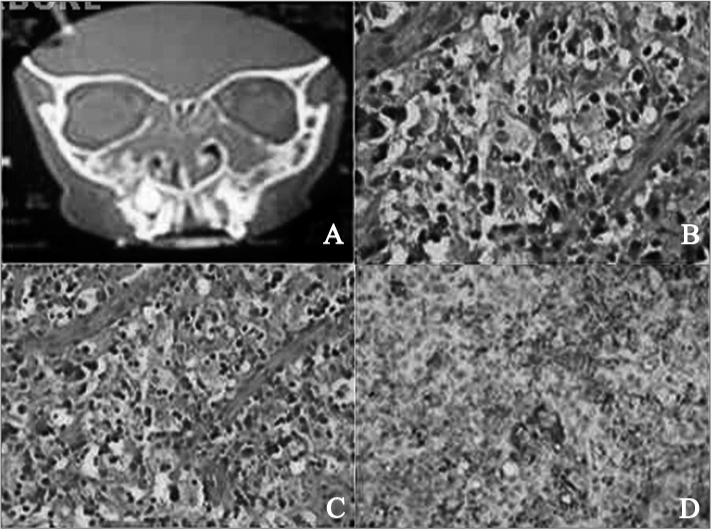
(a) CT scan of the facial sinuses: ethmoidal sinuses filled by soft tissue and maxillary sinuses showing uneven bone thickening in the maxillary walls; (b) and (c) Numerous large histiocytes with eosinophilic cytoplasm (20X or 40X); (d) Immunohistochemistry showing most histiocytes tagged positive for CD68.

L.S.O., male, had ongoing nasal obstruction since the age of 5 and underwent adenotonsillectomy. The symptom persisted and he was prescribed topical steroids. At age 10 the patient suffered from marked worsening of his nasal obstruction, additionally to malnutrition, weight loss, and learning disorders. When he sought help at another center a CT scan of the facial sinuses was ordered and revealed a formation with soft-tissue density occupying the ethmoid cells (obliteration of the intercellular substance) in both maxillary sinuses, along with obstruction and enlargement of the ostiomeatal complexes. The scan showed soft-tissue densities in the nasal concha topography consistent with nose and sinus polyposis (Figure 1). Initial examination of the patient did not reveal lymphadenopathy; nasal endoscopy showed bilateral granulomatous tumors involving the middle meatuses.

The patient was then sent to surgery to have the tumors removed and further tested. During surgery it was observed that the tumors had a brittle, granulomatous aspect resembling histiocytosis. Pathology and immunohistochemistry tests were ordered as the patient was suspected for juvenile xanthogranuloma. Histiocytes, giant cells, and lymphocytes were visualized in the microscope, but immunohistochemistry was positive for CD68 and S100 to confirm the diagnosis of Rosai-Dorfman disease.

The patient was sent to the oncology service, and no systemic involvement was found. He was kept in observation for one year and only then complained of pain in his left leg. MRI scans showed multiple solid nodes in the bone marrow of his lower limbs located predominantly in the patient's left tibia. Such signs suggest bone marrow neoplastic disease consistent with Rosai-Dorfman. The patient underwent chemotherapy for one year and is being currently followed up and seen every six months.

## DISCUSSION AND CONCLUSIONS

RDD occurs more frequently within the first decades of life. Most patients are males (58%)^4^, and people of African descent are affected more predominantly. Our patient was a 10-year-old male of dark complexion.

Diagnosis is verified through immunohistochemistry tests positive for proteins S100 and CD68.

The CT-based diagnosis suggestive of nose and sinus polyposis was questioned from the beginning, as this disease rarely affects children and adolescents^5^.

Juvenile xanthogranuloma was included in the early roster of diagnostic possibilities. This disease must be considered for differential diagnosis purposes, as type II histiocytosis (non-Langerhans cell histiocytosis) occurs during childhood. Males are slightly more affected than females. Microscopic examination of specimens show histiocytes, giant cells, and inflammatory cells. These findings are consistent with RDD, and the difference can be told solely through immunohistochemistry^6^.

There is no consensus in the literature on how to manage RDD patients. Surgery is indicated to improve obstruction-related symptoms.

This case serves as an example of the myriad clinical manifestations seen on RDD patients and illustrates the difficulties in getting to a diagnosis based exclusively on the presence of nasal obstruction. Additionally, it stresses the relevance of considering systemic disease when pediatric patients present symptoms consistent with nose and sinus polyposis.
